# Evaluation of Serum Ischemia Modified Albumin in Patients With COVID-19 Pneumonia: A Case-Control Study

**DOI:** 10.7759/cureus.28334

**Published:** 2022-08-24

**Authors:** Emel Altintas, Ramazan Sabirli, Esra Yuksekkaya, Ozgur Kurt, Aylin Koseler

**Affiliations:** 1 Emergency Medicine, Ankara Training and Research Hospital, Ankara, TUR; 2 Emergency Department, Kafkas University, Kars, TUR; 3 Infectious Disease, Ankara Training and Research Hospital, Ankara, TUR; 4 Department of Microbiology, Acibadem Mehmet Ali Aydinlar University School of Medicine, Istanbul, TUR; 5 Department of Biophysics, Pamukkale University, Denizli, TUR

**Keywords:** covid-19, biomarker, hipoxia, serum ischemia modified albumin, pneumonia

## Abstract

Introduction: Various biomarkers are used when evaluating the hospitalization needs of patients diagnosed with Coronavirus disease (COVID-19). Ischemia-modified albumin (IMA) is a biomarker that causes blood levels to increase as a result of hypoxia and acidosis. We think that an increase in IMA in the blood may be caused by hypoxia stemming from lung damage. This study aimed to compare the mean/median of the blood IMA value in patients with pneumonia due to COVID-19 infection with a control group.

Methods: The case group included patients with COVID-19 pneumonia detected by lung imaging and a positive COVID test. Demographic information of the case group, the severity of pneumonia, and their PCR test results were recorded in the data set.

Findings: A total of 150 people, 90 of whom were in the case group and 60 of whom were in the control group, participated in the study. No statistically significant differences were found between the blood IMA levels of the case group and the control group. When the blood IMA levels of the case group were compared according to pneumonia severity, no statistically significant differences were found between the mild-moderate and severe pneumonia groups.

Conclusion: Blood IMA levels are not a diagnostic biomarker for patients with COVID-19 pneumonia and are not helpful in predicting the severity of pneumonia.

## Introduction

Coronavirus disease (COVID-19), which started in Wuhan, China, in 2019, poses a high risk of transmission, has required hospitalization of patients exceeding hospital capacity in many countries, and can result in mortality [1].

Prognostic markers have an important role in determining the hospitalization needs of patients [1]. Many studies have been conducted on the characteristics of laboratory parameters for COVID-19. COVID-19 is divided into three phases: the early phase, the pulmonary phase, and the hyperinflammation phase [2]. While lymphopenia and thrombocytopenia are seen in the early phase, C-reactive protein (CRP), aspartate aminotransferase (AST), and alanine aminotransferase (ALT) increases are observed in the pulmonary phase. In the hyperinflammation phase, there is a cytokine storm [2]. Increases in CRP, D-dimer, procalcitonin, lactate dehydrogenase (LDH), creatinine, ferritin, brain natriuretic peptide (BNP), and hs-troponin are observed in this phase [2]. These biomarker properties vary according to the progression and severity of COVID-19 [3-4].

Ischemia-modified albumin (IMA) is a negatively charged globular protein at physiological pH [5]. Its blood levels increase as a result of hypoxia and acidosis [3]. As a result of ischemia, acidosis occurs and copper (Cu_2_) is released from proteins and peptides. Cu_2_ binds to the N-terminal region of albumin. Free oxygen radicals damage the Cu_2_ binding site of albumin, and IMA is formed [3]. Pulmonary involvement in patients diagnosed with COVID-19 is used to determine the severity of the disease. We hypothesize that there may be an increase in IMA in the blood due to hypoxia in cases of lung involvement. As such, this study aimed to compare the mean/median of the blood IMA values in patients with pneumonia due to COVID-19 with those in a control group.

## Materials and methods

This study began after receiving the approval of the ethics committee (Pamukkale University-12/11/2020-E.68213). An informed consent form was obtained from the control group individuals, patients, and patients’ first-degree relatives if the participating patients’ level of consciousness was not appropriate to give consent.

Clinical evaluation

Patient Groups

Patients who had symptoms of COVID-19 pneumonia, who were diagnosed with COVID-19 pneumonia, or who had a positive test for COVID-19 according to a reverse transcription polymerase chain reaction (RT-PCR) test administered through nasal swab samples were admitted to the study. The clinical diagnosis and pneumonia severity staging of the patients were made according to the guidelines of the COVID-19 diagnosis and treatment of the Turkish Ministry of Health [1]. The classification of patients with COVID-19 pneumonia was made according to the same guideline.

Mild Moderate Pneumonia

It was defined as patients with symptoms such as fever, muscle/joint pains, cough and sore throat, respiratory rate <30/minute, SpO_2_ level >90% in room air, and mild to moderate pneumonia findings on chest X-ray or computed tomography (CT).

Severe Pneumonia

It was defined as patients with symptoms such as muscle/joint pains, fever, sore throat, and cough, and with clinical findings such as bilateral diffuse pneumonia on chest X-ray or tomography, tachypnea (30/minute), and an SpO_2_ level below 90% in room air.

Control group

Healthy adults in the same sex and age group (±10) as the patients in the case group were included in the control group.

Inclusion Criteria

Inclusion criteria for the patient group included being diagnosed with COVID-19 pneumonia in the emergency department and being at least 18 years old. As for the control group, the criteria included no history of known disease, no use of any medications, and no recent or present infectious symptoms.

Exclusion Criteria

Patients who had a serum albumin level below 3 g/dl and above 5.5 g/dl and who were diagnosed with acute ischemic heart disease/myocardial infarction, trauma, pulmonary embolism, mesenteric ischemia, peripheral vascular disease, acute ischemic cerebravascular disease, muscle diseases, or liver diseases and who did not give written consent were excluded from the study.

Blood Samples

The blood samples taken at the time of admission to the emergency department were centrifuged (3000 rpm, 15 min), their serum was separated, and the serum was stored at −80 °C to be studied.

IMA Level Measurement

Serum IMA levels were measured using commercially available ELISA kits (Human IMA ELISA Kit, MyBioSource, MBS728719, USA) per the manufacturer’s protocols.

Statistical analysis

The SPSS 22.0 Windows program was used for the statistical analysis. Kolmogorov-Smirnov test was used for testing normality distribution. Mann Whitney U tests were conducted to compare two non-parametric, independent groups. p<0.05 level was accepted as the alpha significance level.

## Results

A total of 150 people (90 who have COVID-19 disease and 60 healthy controls), participated in the study. Thirty-six (40%) people in the case group and 27 (45%) people in the control group were female. The mean ages of the case group and controls were 66.1±14.7 and 59.1±15.5 years, respectively.

The median serum IMA level was 5.1 (3.4-7.9) IU/mL in the COVID-19 patient group, while it was found to be 4.6 (3.32-7.07) IU/mL in the control group. No statistically significant differences were found between the blood IMA levels of the case group and the controls (p=0.446) (Table 1).

**Table 1:** Serum ischemia-modified albumin levels IMA: ischemia-modified albumin, IQR: interquartile range

Serum IMA levels in patient and control groups	IMA levels (IU ml)
COVID-19 patients, n=90 median, IQR	5.1 (3.4–7.9)
Controls, n=60, median, IQR	4.6 (3.32–7.07)
P-value	0.446
Serum IMA levels in patients’ subgroups	
Mild-to-moderate pneumonia n=57, median, IQR	5 (3.4–7.4)
Severe pneumonia n=33, median, IQR	5.1 (3–10.4)
P-value	0.700

When the case group was examined as two groups according to the pneumonia severity level of the patients, mild-to-moderate and severe pneumonia, no statistically significant differences were found between these groups in terms of serum IMA levels (p=0.7) (Table 1).

## Discussion

The pulmonary involvement of COVID-19 patients has been explained by different mechanisms. Although they share a single etiology (SARS-CoV-2), these severely hypoxemic patients can be quite different from one another [6]. Viral infection causes moderate local subpleural interstitial edema where stress and strain are concentrated, especially at the interfaces between lung structures with different elastic properties. Edema, which increases over time, has been reported to increase lung weight, overlapping pressure, and dependent atelectasis [6].

In another study examining perfusion changes in patients with COVID-19 pneumonia, three main findings were obtained, including increased perfusion proximal to areas of lung opacity; decreased peripheral perfusion areas corresponding to peripheral lung opacities; and increased perfusion halo consolidation surrounding peripheral areas. These perfusion abnormalities, combined with pulmonary vascular enlargement, suggest intrapulmonary shunting towards areas of impaired gas exchange, resulting in clinical hypoxia and worsening ventilation-perfusion mismatch. Although hypo-perfused peripheral opacities are also seen in pulmonary embolism and pulmonary infarction, as in pulmonary embolism or pulmonary infarction, segmental perfusion increase is not observed in pulmonary opacities in COVID-19 disease. Therefore, opacities seen in COVID-19 disease are stated as atypical [7].

This study found no differences in the blood IMA levels of patients with mild-moderate and severe COVID-19 pneumonia. Blood IMA levels may not have increased in COVID-19 patients due to a number of factors such as pulmonary involvement of COVID-19 patients ranging from mild to severe; complete obstruction due to ischemic process; hypoperfusion and thrombus were not in the foreground; interstitial pulmonary edema as a result of increased lung permeability due to the inflammatory process; worsening of this condition with intrapulmonary shunt, and pulmonary embolism; and different characteristics from perfusion abnormalities were exhibited.

A study evaluating oxidative stress biomarkers stated that IMA level predicted severity and intensive care hospitalization in COVID-19 patients [8]. In the study, the COVID-19 severity classification was made according to the NIH classification (mild, moderate, severe, and critical). Patients without tomography findings in the mild class were included [9]. In the study, it is observed that there is a significant difference between the mild class and the other classes. In another study, COVID-19 patients with and without lung involvement were compared, and it was stated that IMA level could be a predictive factor in lung involvement [10]. In our study, COVID-19 was divided into two classes as mild-moderate and severe, and patients with lung involvement in both classes were included. Due to this classification difference, we think there is no difference between mild-moderate and severe classes.

In another study, it was stated that IMA levels were higher in the early phase of COVID-19 than in the acute phase [11]. One of the limitations of our study is that the days of the COVID-19 patients were not included. Other limitations of our study are that although the control group was selected from healthy people, it was thought that serum IMA measurement in healthy people could be an influencing factor, especially in the use of antioxidant drugs and hormone therapy in menopausal women [12-13]. A small number of patients in the subgroup of the COVID-19 group and only IMA were studied in our study. We think that studies with larger samples and comparing COVID-19 subgroups with IMA and other biomarkers are needed.

## Conclusions

In our study, blood IMA levels are not a diagnostic biomarker for patients with COVID-19 pneumonia and are not helpful in predicting the severity of pneumonia. We think that it is important to classify the COVID-19 mild-moderate and severe groups and on which day the serum IMA level is studied after the symptoms of COVID-19.

We think that studies with larger samples and comparing COVID-19 subgroups with IMA and other biomarkers are needed. We think that meta-analysis studies can be done by evaluating according to COVID severity groups and whether there is an early phase or not.
